# Differences in attenuation pattern in myocardial SPECT between CZT and conventional gamma cameras

**DOI:** 10.1007/s12350-018-1296-6

**Published:** 2018-05-23

**Authors:** Jenny Oddstig, Elin Martinsson, Jonas Jögi, Henrik Engblom, Cecilia Hindorf

**Affiliations:** 1 Department of Radiation Physics, Skåne University Hospital, Lund, Sweden; 2 Department of Medical Radiation Physics, Clinical Sciences, Lund University, Lund, Sweden; 3 Department of Clinical Physiology and Nuclear Medicine, Skåne University Hospital, Lund University, 221 85 Lund, Sweden

**Keywords:** Myocardial perfusion imaging, SPECT, CZT detector, conventional gamma camera, attenuation artifact

## Abstract

**Background:**

In myocardial perfusion imaging (MPI), single-photon emission tomography (SPECT) soft-tissue attenuation by the abdomen, breasts, and lateral chest wall may create artifacts that mimic true perfusion defects. This may cause misdiagnosis of myocardial perfusion. The aim of the present study was to compare the localization, extent, and depth of attenuation artifacts in MPI SPECT for a multi-pinhole cadmium zinc telluride (CZT) camera vs a conventional gamma camera.

**Methods:**

Phantom and patient measurements were performed using a CZT camera (GE NM 530c) and a conventional gamma camera (GE Ventri). All images were attenuation corrected with externally acquired low-dose computed tomography. The localization, extent, and depth of the attenuation artifact were quantified by comparing attenuation-corrected and non-attenuation-corrected images.

**Results:**

Attenuation artifacts were shifted from the inferolateral wall to the lateral wall using the CZT camera compared to a conventional camera in both the patient and the phantom. The extent of the attenuation artifact was significantly larger for the CZT camera compared to the conventional camera (23 ± 5% vs 15 ± 5%, *P* < .001) for patients and the result was similar for the phantom (28% vs 19%). Furthermore, the depth of the attenuation artifact (percent of maximum counts) was less pronounced for the CZT camera than for the conventional camera, both for phantom measurements (73% vs 67%) and patients (72 ± 3% vs 68 ± 4%, *P* < .001).

**Conclusions:**

Attenuation artifacts are found in different locations to different extents and depths when using a CZT camera vs a conventional gamma camera for MPI SPECT. This should be taken into consideration when evaluating MPI SPECT studies to avoid misinterpretation of myocardial perfusion distribution.

**Electronic supplementary material:**

The online version of this article (10.1007/s12350-018-1296-6) contains supplementary material, which is available to authorized users.

## Introduction

Myocardial perfusion imaging (MPI) with single-photon emission computed tomography (SPECT) is the most widely used non-invasive imaging method for the detection of stress-induced ischemia and viability due to flow-limiting coronary stenosis. Traditionally, conventional MPI SPECT images have been acquired using a scintillation gamma camera with a parallel-hole collimator and a rotating gantry. Recently, a new generation of dedicated gamma cameras for MPI with cadmium zinc telluride (CZT) detectors, stationary gantry, and multi-pinhole collimators has been introduced. The agreement between multi-pinhole CZT technology and conventional technology using a NaI-crystal for visualizing myocardial perfusion has been clinically validated in several studies.^1^–^4^ CZT technology is associated with higher spatial resolution, higher sensitivity, and better energy resolution compared to conventional gamma cameras with NaI-crystals.^5^–^7^ The CZT camera system also enables complete 3D coverage of the heart using a stationary gantry. This permits sampling of the total heart volume simultaneously in only a few minutes and/or reducing the administered activity.^8^,^9^

The diagnostic accuracy for MPI is high, and the use of MPI SPECT for risk stratification has been well-validated.^10^,^11^ However, localized soft-tissue attenuation by the abdomen, breasts, and lateral chest wall may create artifacts that mimic true perfusion defects. Diagnostic specificity for coronary artery disease can be increased if attenuation correction is applied to MPI SPECT images. Attenuation correction of MPI SPECT studies for the CZT camera using X-ray-based computed tomography (CT) has been compared to attenuation-corrected MPI SPECT for conventional dual-head cameras.^12^–^14^ The results indicate that attenuation correction (AC) of MPI SPECT for CZT cameras gives the same result as that for a conventional gamma camera. However, most dedicated CZT cameras for MPI are not equipped with an integrated CT component, which is why many MPI studies using CZT cameras are performed without attenuation correction. When MPI studies are interpreted without attenuation correction, it is important to be aware of the typical attenuation pattern in order to prevent attenuation artifacts from being interpreted as perfusion abnormalities.

To what extent the attenuation patterns for the CZT camera with pinhole collimators and static gantry differ from the conventional gamma camera with parallel-hole collimators and rotating gantry is not known. Therefore, the aim of this study was to compare the localization, extent, and depth of attenuation artifacts in myocardial perfusion SPECT both in phantom experiments and in patients using the two different gamma cameras.

## Materials and Methods

### Phantom Measurements

Phantom measurements were performed on both a CZT gamma camera (Discovery NM 530c; GE Healthcare, Milwaukee, WI, USA) and a conventional gamma camera (Ventri; GE Healthcare, Milwaukee, WI, USA) using an elliptical lung-spine body phantom model ECT/LUNG/P with the cardiac insert model ECT/CAR/I (Data Spectrum Corporation, Durham, NC, USA). The myocardial wall of the left ventricle in the cardiac insert was filled with 0.25 MBq/mL (0.0066 mCi/mL) ^99m^Tc. The torso was filled with water and positioned in the gamma cameras using an external laser to obtain a repeatable position. For the CZT camera, positioning was performed to ensure that the heart was placed in the center of the camera’s field of view. The phantom was imaged with both cameras with an acquisition time to obtain at least twice the number of acquired counts compared to clinical patient images. The phantom measurements were first performed with the cardiac insert homogenously filled with ^99m^Tc. Thereafter, measurements were repeated using both cameras with a 2.1-cm^3^ solid defect situated centrally in the anterior wall. CT for attenuation correction of the phantom images was performed using an external CT (GE Discovery PET/CT 690; GE Healthcare, Milwaukee, WI, USA).

### Patient Measurements

Twenty-two patients (five females) with suspected stable ischemic heart disease were included in the study. Patient characteristics are shown in Table 1. The patients were examined using a 2-day stress-rest protocol. The stress was accomplished by an adenosine infusion, and the patients received an intravenous injection of 4 MBq/kg (0.11 mCi/kg) body weight of ^99m^Tc-tetrofosmin in both stress and rest. All patients were examined in a supine position with the arms over the head using both the CZT and conventional camera approximately 1 hour after administration of ^99m^Tc-tetrofosmin. A CT for attenuation correction of the MPI SPECT images was obtained (Discovery PET/CT 690; GE Healthcare, Milwaukee, WI, USA) on the same day that the stress examination was performed. In total, 74 acquisitions were performed. Nineteen patients were examined using both cameras during the stress test, and 18 were examined with both cameras at rest. The study was approved by the regional ethics committee, and all patients gave their written informed consent to participate in the study.

**Table 1:** Patient characteristics (N or mean ± SD)

	Study population *n* = 22
Age years	69 ± 6.4 (59–87)
Male	17 (77%)
Female	5 (23%)
BMI (kg/m^2^)	27 ± 3.2 (21–35)
Body weight (kg)	84 ± 11 (62–101)

### Image Acquisition and Reconstruction

The acquisition time on the CZT camera was 480 seconds for both stress and rest. The images were reconstructed with the Maximum Likelihood Estimation Method (MLEM) algorithm using 40 iterations (Green OSL regularization alpha parameter of 0.51 and a beta of 0.3 followed by post-filtering with a Butterworth filter with a cut-off frequency of 0.37 cycles/cm and a power of 7). For the CZT camera, the voxel size was 4 × 4 × 4 mm.

For the conventional camera, an examination was performed with the detectors in L-mode. Sixty projections were acquired at a total angular range of 180° with a stop condition of 25 seconds per projection for both stress and rest. The images on the conventional gamma camera were reconstructed with a resolution recovery OSEM algorithm (Evolution, GE Healthcare) using 12 iterations and 10 subsets. This was post-filtered with a Butterworth filter with a cut-off frequency of 0.4 and a power of 10. All reconstruction parameters used followed the recommendations from the manufacturer. The voxel size for the conventional camera was 6.4 × 6.4 × 6.4 mm.

All images were reconstructed both without attenuation correction and with attenuation correction using the externally acquired CT. The reconstructed images were reformatted to the standard cardiac axis format (short axis, vertical long axis, and horizontal long axis).

### Image Analysis

**Figure 1:**
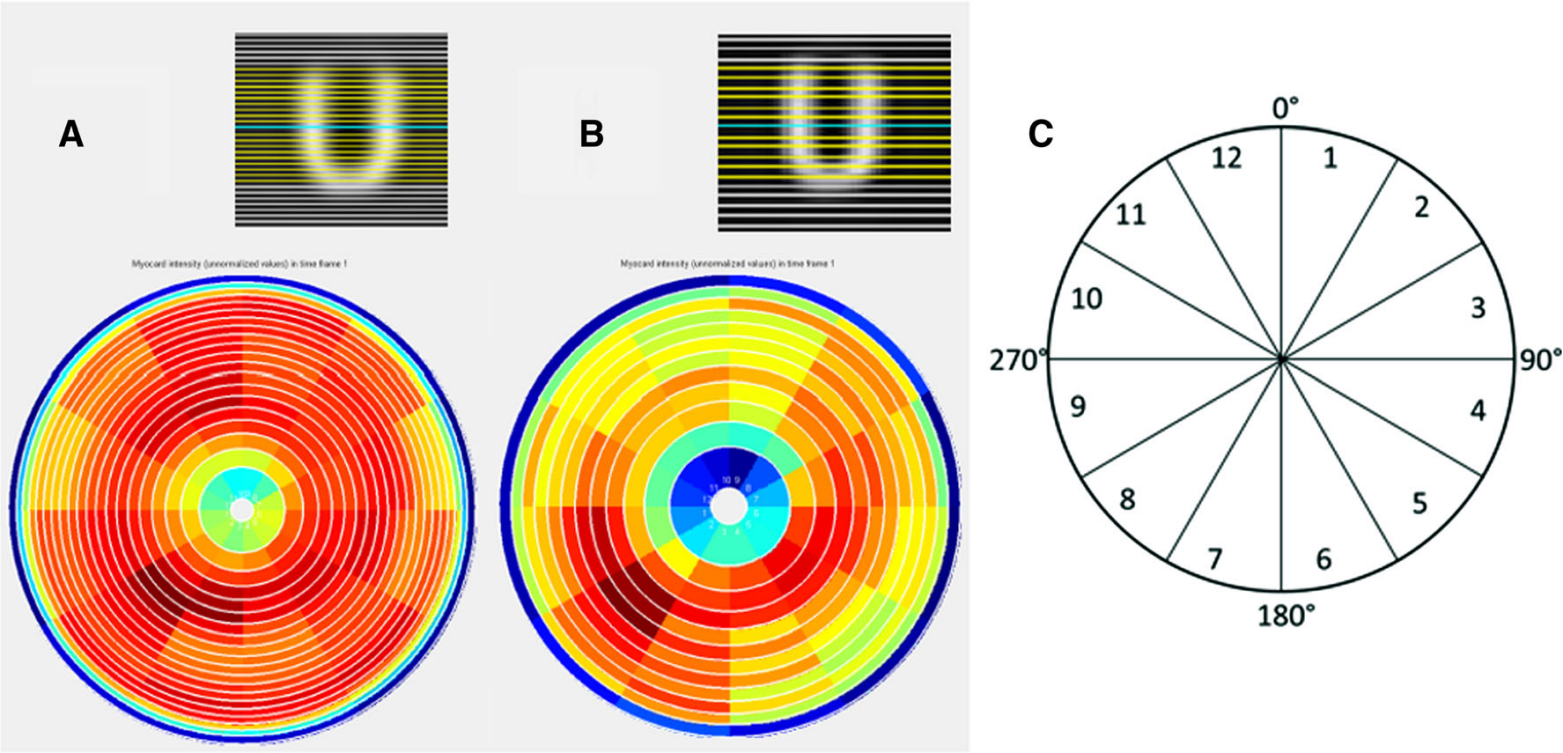
The segmentation of the left ventricle in **A** the multi-pinhole cadmium zinc telluride (CZT) camera with the heart volume in 20 short-axis slices, **B** the conventional camera with the heart volume in 12 short-axis slices, and **C** each short-axis slice of the left ventricle divided into 12 sectors

All data were analyzed using the software Segment v 2.0.^15^ The left ventricle was segmented from the short-axis images. A fixed number of slices counted from the base (20 for the CZT and 12 for the conventional camera, depending on the voxel thickness) was analyzed. Each slice was divided into 12 equally sized sectors. The number of counts in each segment was exported to Excel and normalized to the total number of counts (Figure 1).

**Figure 2:**
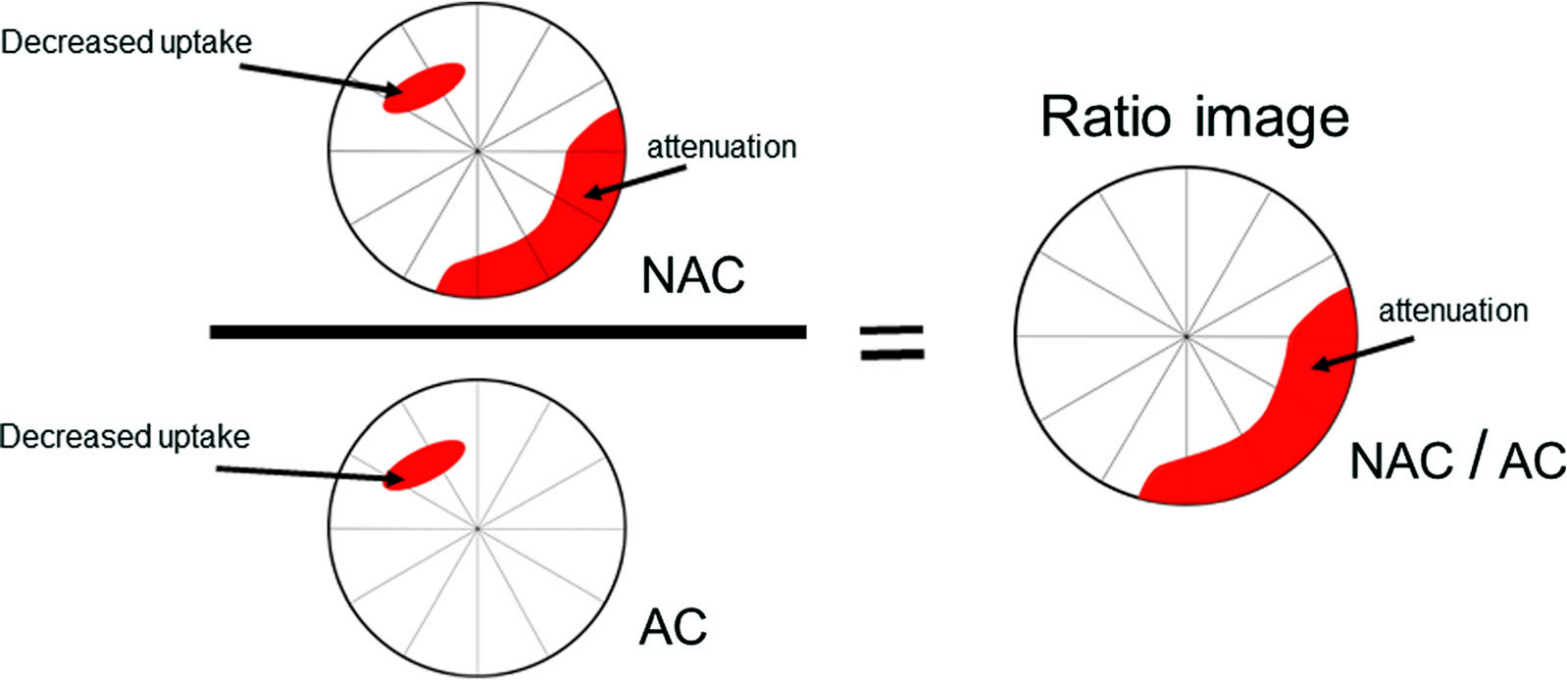
The ratio image of a non-attenuation-corrected (NAC) image and the attenuation-corrected (AC) image characterizing the attenuation artifacts, showing that all sectors not containing attenuation have a ratio of approximately 1, and the sectors with attenuation have a value < 1

The ratio of the non-attenuation-corrected (NAC) image to the attenuation-corrected (AC) image from the same examination was calculated for each patient using both cameras. The same was done for the phantom measurements. The ratio was used as a descriptor of the attenuation artifacts. For all sectors not containing any attenuation, the ratio was 1, and sectors with attenuation had a ratio below 1 (Figure 2).

From the ratio images of the phantom, a threshold for delineation of attenuation was defined. The threshold was specified by experienced observers by finding the value at which the attenuation artifact was included, but nothing more. These thresholds, which were specific for each gamma camera, were used on NAC/AC-ratio images in patients and phantoms to determine the extent, localization, and depth of the attenuation artifact.

**Figure 3:**
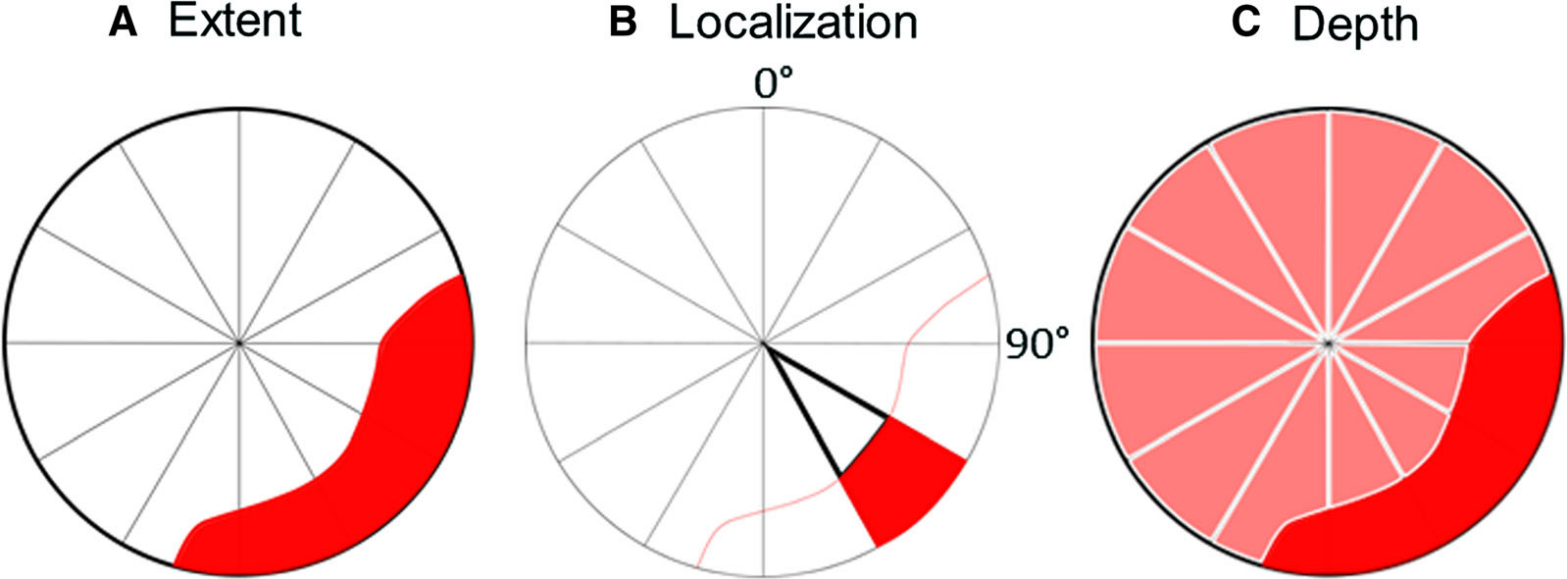
Three different parameters defining differences in the attenuation artifact were used to show that **A** the extent of attention artifact is the fraction of the image containing the attenuation, **B** the localization is centrum sector containing the attenuation, and **C** the depth is the reduction of counts in the area with attenuation

The extent of the attenuation artifact was determined as the number of segments in which the attenuation artifact was present divided by the total number of segments (Eq. 1). The extent was considered to be the fraction of the image containing the attenuation (Figure 3A).

1 $$ {\text{Extent}} = \frac{{{\text{Number}}\;{\text{of}}\;{\text{segment}}\; {\text{in}}\; {\text{the}} \;{\text{attenuation}}}}{{{\text{Total }}\;{\text{number }}\;{\text{of}} \;{\text{segments}}}} $$

The localization of attenuation was defined as the angle of the attenuation artifact center according to the reference system described in Figure 3B. Furthermore, the depth of attenuation was calculated as the mean of counts in the attenuation segments divided by the mean of counts in the 30% of segments with the highest counts (Eq. 2). The depth was considered to be the reduction of counts in the area with attenuation (Figure 3C).

2 $$ {\text{Depth}} = \,\frac{{{\text{mean}} \;{\text{counts}}\; {\text{in}} \;{\text{the}}\; {\text{attenuation}} \;{\text{segments}}}}{{{\text{mean}}\; {\text{of}}\; {\text{counts}}\; {\text{in}}\; {\text{the}} \;30 \%  \;{\text{of}}\; {\text{segment }}\;{\text{with}}\; {\text{highest}} \;{\text{counts}}}}. $$

### Statistical Analysis

The data are presented as mean ± standard deviation (SD). A paired *t* test was used to test for differences between the two cameras. Statistical significance was defined as *P* < .05.

## Results

### Attenuation Thresholds

**Figure 4:**
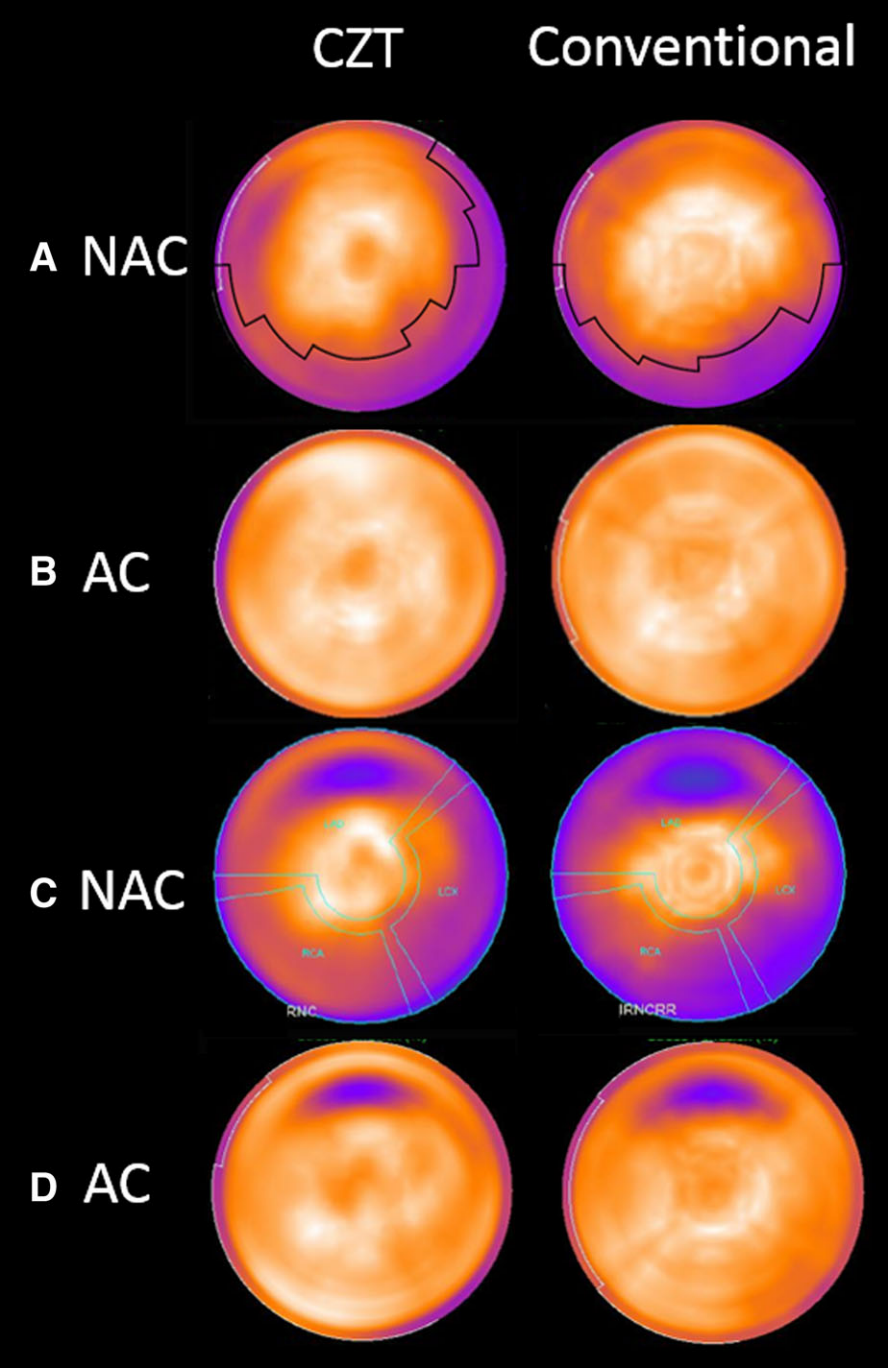
The polar plots of the phantom measured in the multi-pinhole cadmium zinc telluride (CZT) and conventional camera showing **A** non-attenuation-corrected images with the threshold value of the attenuation artifact marked with a solid line where the attenuation artifact has different patterns on the camera with different techniques, **B** CT attenuation-corrected (AC) images where the attenuation artifact cannot be seen, **C** a 2.1-cm^3^ defect situated in the anterior wall where the defect appears in the same area on both cameras while the attenuation artifact has different patterns, and **D** CT attenuation-corrected (AC) images of the phantom with a 2.1-cm^3^ defect in the anterior wall

The threshold for delineation of attenuation was determined to be 0.90 and 0.85 for the CZT and conventional camera, respectively. The delineation of attenuation in the phantom experiment for both cameras is shown in Figure 4A. Attenuation correction with CT produced similar images of the phantom for the two cameras (Figure 4B). The phantom measurements with a defect in the anterior wall confirmed the attenuation shift, yielding a defect located in the same sector for both cameras, while the attenuation artifact was shifted (Figure 4C).

### Attenuation Localization

The phantom measurements showed a shift in attenuation from the inferolateral wall to the lateral wall for the CZT camera compared to the conventional camera. The calculation of the localization of the attenuation artifacts showed a shift of 15° and 13° from the inferolateral wall to the lateral wall for the CZT camera compared to the conventional camera, respectively (Table 2). The difference between the cameras was statistically significant in patients (*P* < .001).

**Table 2:** The mean localization, extent, and depth of the attenuation artifact for phantom and patient

	CZT camera	Conventional camera
Localization		
Phantom	150°	165°
Patient mean	155° ± 17°	168° ± 15°
Extent		
Phantom	28%	19%
Patient mean	23 ± 5%	15 ± 5%
Depth		
Phantom	73%	67%
Patient mean	72 ± 3%	68 ± 4%

The difference between the cameras was statistically significant in patients (*P* < .001)

### Attenuation Extent

The extent of attenuation for the phantom experiment was larger in the CZT images compared to the conventional images (28% and 19%, respectively). The corresponding value of the extent of attenuation in the acquisitions of 74 patients was 23 ± 5% and 15 ± 5% for the CZT images and the conventional images, respectively (*P* < .001).

### Attenuation Depth

For the phantom measurements, the depth (percent of maximum counts) within the attenuation artifact was 73% and 67% for the CZT camera and conventional gamma camera, respectively. The corresponding value of the depth of the attenuation artifact for the 74 patient acquisitions was 72 ± 3% for the CZT camera and 68 ± 4% for the conventional camera (*P* < .001).

**Figure 5:**
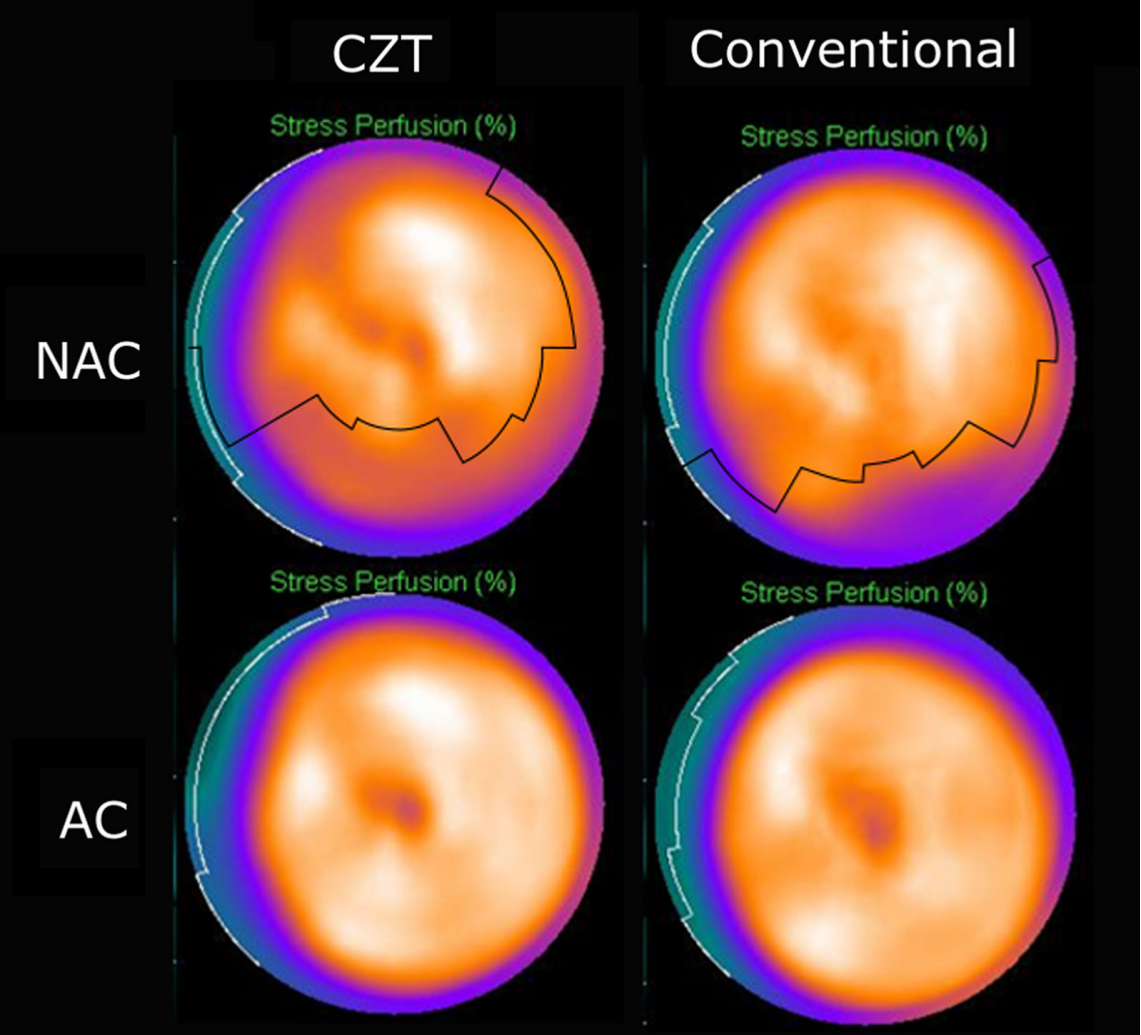
The NAC and AC bullseye plot images for one patient examined using both the multi-pinhole cadmium zinc telluride (CZT) camera and the conventional camera, showing the threshold value of the attenuation artifact marked with a solid line in the NAC images

Figure 5 shows an example of one patient examined with both the CZT camera and the conventional gamma camera.

## Discussion

This study characterized the differences in the attenuation artifact for the CZT camera compared to a conventional gamma camera. The attenuation artifact was shifted counter-clockwise from the inferolateral wall to the lateral wall for the CZT camera. The attenuation artifact was distributed over a larger part of the image, but attenuation was less deep for the CZT camera compared to the conventional camera. The difference in attenuation localization was not caused by variations in phantom positioning because a shifted attenuation artifact also appeared in the phantom measurements with a defect, and the defect was located in the same position for both cameras. This also showed that the shift in the attenuation artifact was not an effect of the different linearization of the myocardium in the reconstruction. Both the phantom and the patients were accurately positioned within the camera’s field of view to avoid positioning defects that have been shown to occur with the CZT camera under suboptimal positioning.^16^

The difference in attenuation artifact patterns between the CZT and conventional gamma camera may arise from the difference in the measurement geometry. Using a parallel-hole collimator in the conventional camera, the detected photons are emitted perpendicular to the detector for all pixels in all 60 projections at 180° around the patient. The CZT camera measures the object with 19 stationary pinhole collimators arranged in three rows. The detectors in the central row are directed at the same angle as that for the conventional camera with the parallel-hole collimator. The detectors located on each side of the central row are rotated against the center of the detected volume. The use of pinhole collimators and the rotated detectors produces a different image for the CZT camera compared to the parallel-hole collimator. It also generates other distance angles with respect to the myocardium, which is why the attenuation artifacts may occur to different extents. The parallax error for the pinhole collimators may also be a contributing factor for the difference in the attenuation artifact patterns between the cameras.

The use of CTs for attenuation correction (AC) maps from another scanner has previously been useful for attenuation correction of MPI SPECT as long as the co-registration of all image stacks was previewed.^17^–^19^ The same result was obtained for the CZT camera and for the conventional cameras. The NAC/AC ratio can therefore be used to analyze the difference in attenuation artifact patterns obtained using the different cameras.

The calculation for the relative depth for the phantom measurement concluded that the attenuation artifact was deeper in the conventional camera compared to the CZT camera (67% and 73%, respectively). The patient measurements correlated well with the phantom results (68 ± 4% and 72 ± 3% [*P* < .05] for the conventional and CZT cameras, respectively). The results in the current study show less isotope reduction in the attenuation artifact for the CZT camera compared to the conventional gamma camera, which supports the findings of Liu et al.^20^

The extent of the attenuation artifact differed between the CZT camera and the conventional camera for both the phantom measurements and those in patients. Thus, it is important for physicians interpreting MPI images to be aware of the differences in attenuation patterns for conventional and CZT pinhole gamma cameras when evaluating non-attenuation-corrected images so that they do not overestimate uptake reductions in the lateral wall or underestimate uptake reductions in the inferolateral wall, producing false-positive or false-negative results.

## Limitations

Due to the predominant number of males in the patient population with mostly inferior wall attenuation, it is not possible to draw any conclusions regarding the anterior attenuation artifacts arising in women as a result of breast attenuation. The clinical impact of these findings has not been studied here because not all patients were examined using both cameras under both stress and rest conditions. Thus, this report should be considered a proof-of-concept study of different attenuation patterns for CZT cameras compared to conventional cameras. Differences between patients and phantoms might be affected by basing the threshold on experienced observer opinions. This was, however, the only way to approach this study given the lack of any true reference standard. The use of mean counts over a large area might lessen the impact of more focal attenuation artifacts; however, this results in a method not influenced by an observer.

## New Knowledge Gained

This study establishes differences in the appearance of inferior attenuation artifacts for the CZT camera (GE Discovery NM 530c) compared to the conventional gamma camera (GE Ventri). It is important to consider these differences when interpreting data and especially when comparing images from different cameras so that one does not over- or underestimate perfusion defects in the myocardium.

## Conclusions

Attenuation artifacts are produced in different locations to varying extents and depths when comparing a multi-pinhole cadmium zinc telluride (CZT) camera to a conventional gamma camera for MPI SPECT. This needs to be taken into consideration when evaluating MPI studies to avoid misinterpreting the location and extent of a myocardial perfusion distribution.

## Electronic supplementary material

Below is the link to the electronic supplementary material.

Supplementary material 1 (PPTX 1915 kb)

## Abbreviations

AC: Attenuation corrected
BMI: Body mass index
CT: Computed tomography
CZT: Multi-pinhole cadmium zinc telluride
MLEM: Maximum likelihood estimation method
MPI: Myocardial perfusion imaging
NAC: Non-attenuation corrected
OSEM: Ordered subset expectation maximization
SD: Standard deviation
SPECT: Single-photon emission computed tomography
