# Supplementary material for: Differences in attenuation pattern in myocardial SPECT between CZT and conventional gamma cameras
Source: J Nucl Cardiol. 2018 May 23;26(6):1984–91. doi: 10.1007/s12350-018-1296-6 (PMC6908561; doi:10.1007/s12350-018-1296-6)
Supplement: Supplementary file 1 — Supplementary material 1 (PPTX 1915 kb) [file 12350_2018_1296_MOESM1_ESM.pptx]

## Slide 1
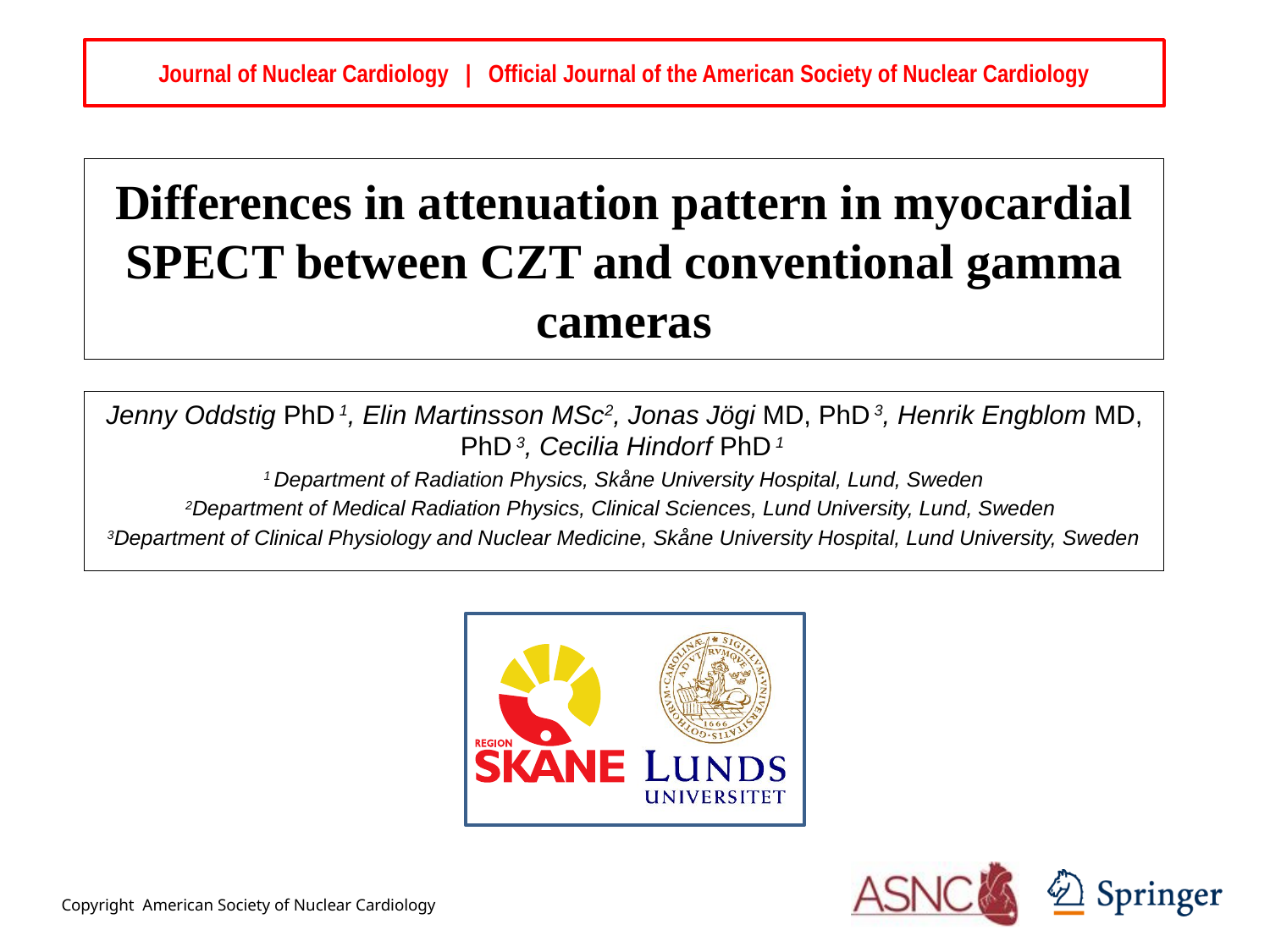

Journal of Nuclear Cardiology | Official Journal of the American Society of Nuclear Cardiology
# Differences in attenuation pattern in myocardial SPECT between CZT and conventional gamma cameras
Jenny Oddstig PhD 1, Elin Martinsson MSc2, Jonas Jögi MD, PhD 3, Henrik Engblom MD, PhD 3, Cecilia Hindorf PhD 1
1 Department of Radiation Physics, Skåne University Hospital, Lund, Sweden
2Department of Medical Radiation Physics, Clinical Sciences, Lund University, Lund, Sweden
3Department of Clinical Physiology and Nuclear Medicine, Skåne University Hospital, Lund University, Sweden
Copyright American Society of Nuclear Cardiology

## Slide 2
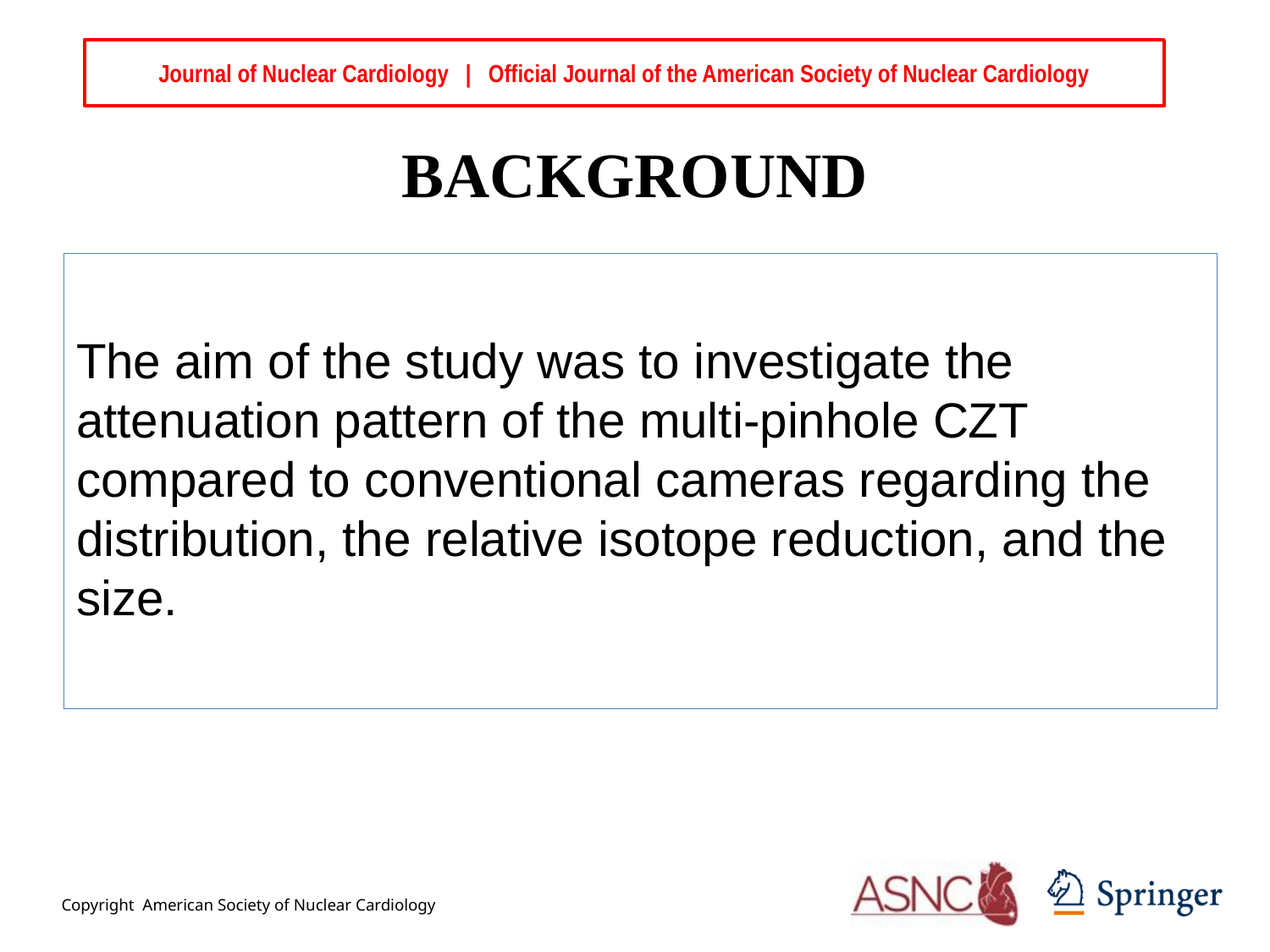

Journal of Nuclear Cardiology | Official Journal of the American Society of Nuclear Cardiology
# BACKGROUND
The aim of the study was to investigate the attenuation pattern of the multi-pinhole CZT compared to conventional cameras regarding the distribution, the relative isotope reduction, and the size.
Copyright American Society of Nuclear Cardiology

## Slide 3
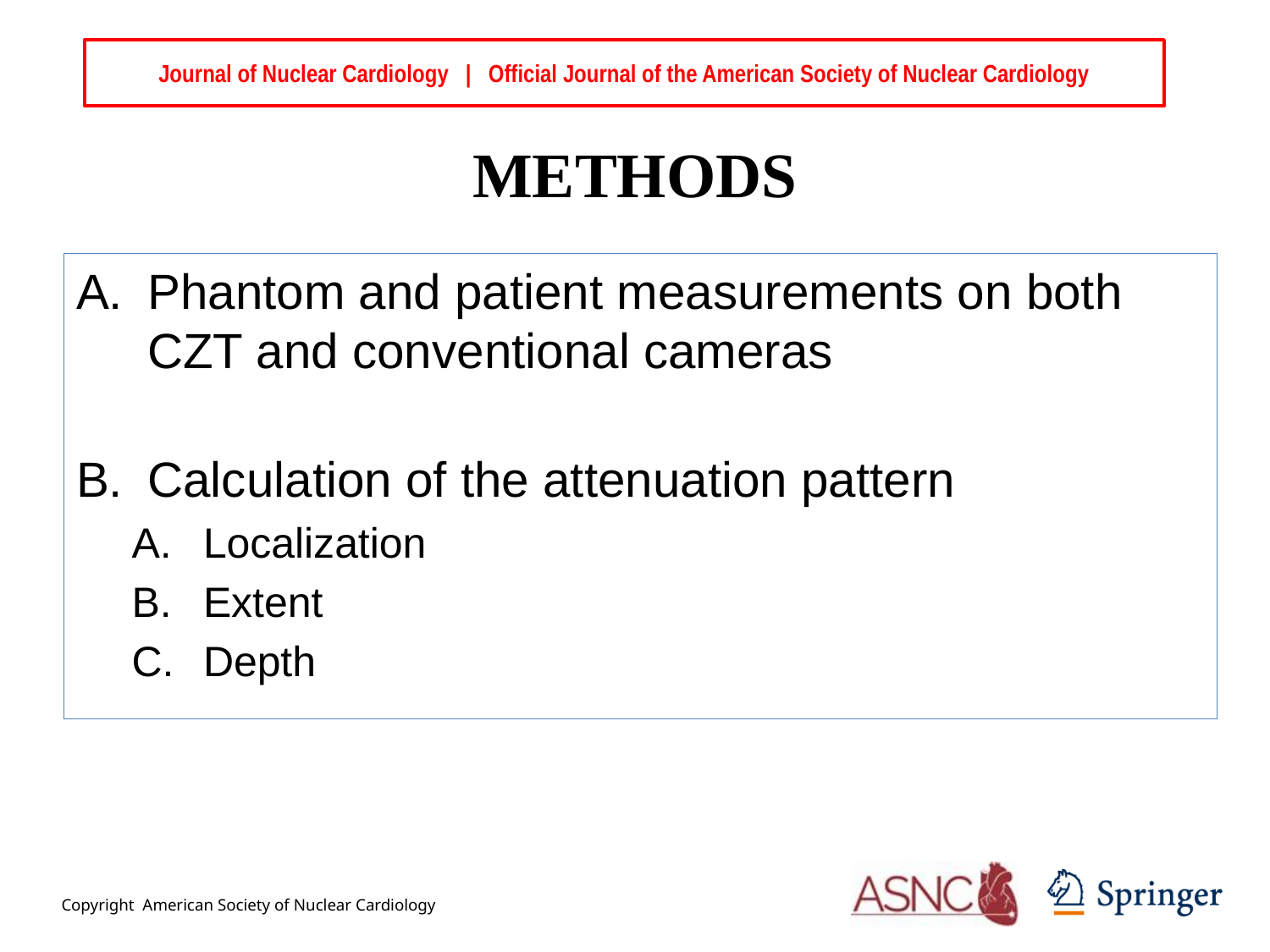

Journal of Nuclear Cardiology | Official Journal of the American Society of Nuclear Cardiology
# METHODS
Phantom and patient measurements on both CZT and conventional cameras
Calculation of the attenuation pattern
Localization
Extent
Depth
Copyright American Society of Nuclear Cardiology

## Slide 4
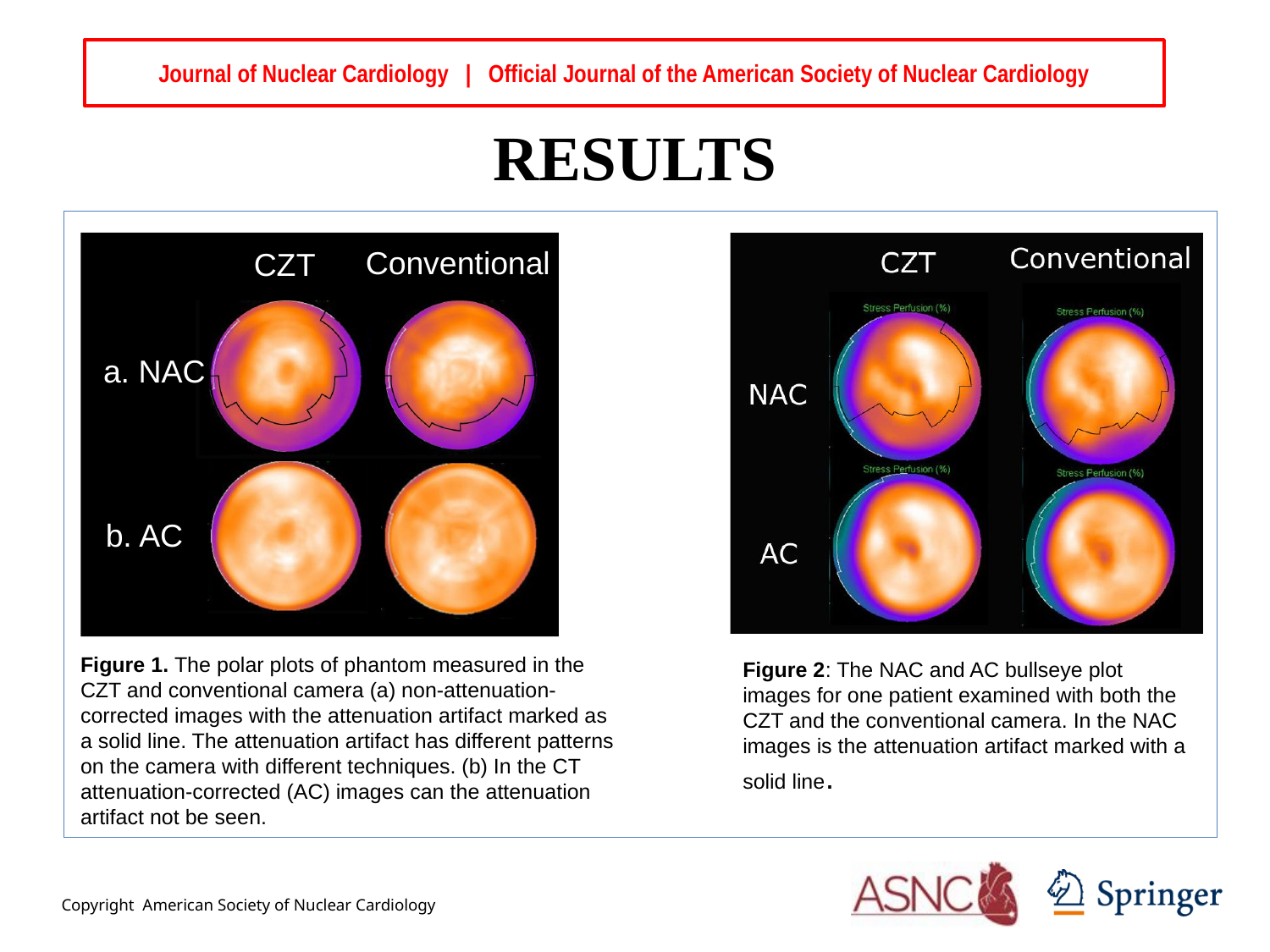

Journal of Nuclear Cardiology | Official Journal of the American Society of Nuclear Cardiology
# RESULTS
a.
a. NAC
b. AC
CZT
Conventional
Figure 1. The polar plots of phantom measured in the CZT and conventional camera (a) non-attenuation-corrected images with the attenuation artifact marked as a solid line. The attenuation artifact has different patterns on the camera with different techniques. (b) In the CT attenuation-corrected (AC) images can the attenuation artifact not be seen.
Figure 2: The NAC and AC bullseye plot images for one patient examined with both the CZT and the conventional camera. In the NAC images is the attenuation artifact marked with a solid line.
Copyright American Society of Nuclear Cardiology

## Slide 5
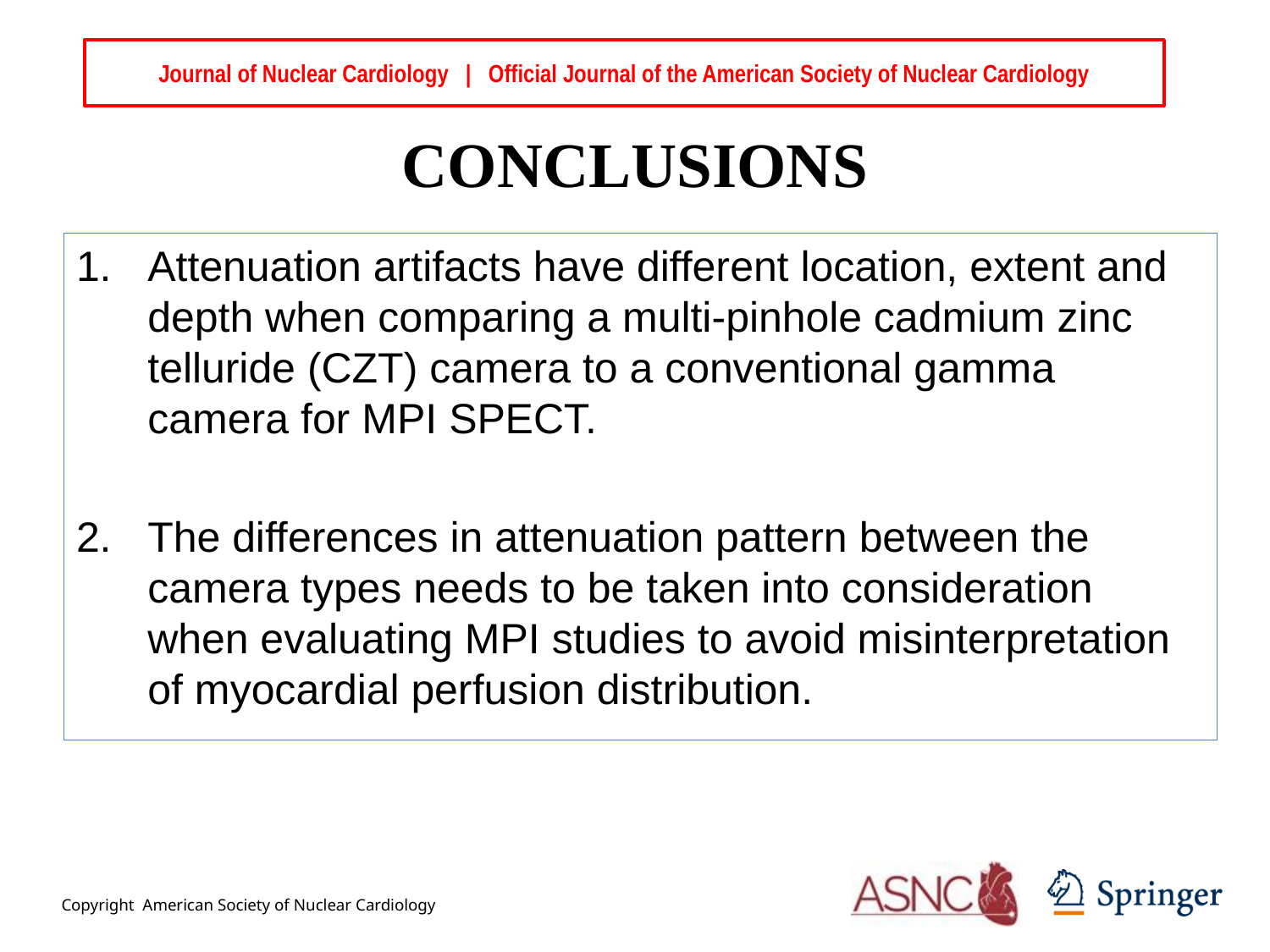

Journal of Nuclear Cardiology | Official Journal of the American Society of Nuclear Cardiology
# CONCLUSIONS
Attenuation artifacts have different location, extent and depth when comparing a multi-pinhole cadmium zinc telluride (CZT) camera to a conventional gamma camera for MPI SPECT.
The differences in attenuation pattern between the camera types needs to be taken into consideration when evaluating MPI studies to avoid misinterpretation of myocardial perfusion distribution.
Copyright American Society of Nuclear Cardiology
